# rs10010131A in *WFS1* is Associated with Elevated Serum Urea in Type 2 Diabetes Patients Treated with SGLT2 Inhibitors

**DOI:** 10.2147/TCRM.S578286

**Published:** 2026-04-28

**Authors:** Khaled Naja, Asma A Elashi, Laila Hedaya, Najeha Rizwana Anwardeen, Mashael Al-Shafai, Manfredi Rizzo, Mohamed A Elrayess

**Affiliations:** 1 Biomedical Research Center, QU Health, Qatar University, Doha, Qatar; 2 Department of Biomedical Sciences, College of Health Sciences, QU Health, Qatar University, Doha, Qatar; 3 School of Medicine, Promise Department, University of Palermo, Palermo, Italy; 4 Department of Medicine, Ras Al Khaimah (RAK) Medical and Health Sciences University, Ras Al Khaimah, United Arab Emirates; 5 College of Medicine, QU Health, Qatar University, Doha, Qatar

**Keywords:** sodium-glucose cotransporter 2 inhibitors, metformin, type 2 diabetes, urea, wolframin, pharmacogenomics

## Abstract

**Introduction:**

Sodium-glucose cotransporter 2 inhibitors (SGLT2i) are effective agents for type 2 diabetes (T2D) management, yet interindividual variability in response mechanisms remains unclear. This study examined genetic interactions influencing clinical and biochemical outcomes among SGLT2i treated patients.

**Methods:**

Data from 13,808 Qatar Biobank participants were analyzed, including 207 propensity score–matched T2D patients stratified into three groups: SGLT2i-treated, metformin monotherapy, and drug-naïve. Significant clinical traits across groups were further assessed for genotype effects within SGLT2i treated patients.

**Results:**

Compared with both comparators, SGLT2i-treated individuals showed elevated serum urea (FDR < 0.05). Genetic analysis identified an association between the *WFS1* rs10010131 A allele and higher urea levels exclusively in SGLT2i treated patients (β = +0.63 mmol/L per A allele, 95% CI [0.14–1.11], p = 0.012). This variant showed strong linkage disequilibrium *(r^2^* = 0.95, D′ = 0.99) with rs6446482 in *WFS1*. Kidney-specific eQTL data revealed reduced *WFS1* expression in A-allele carriers.

**Discussion:**

These findings suggest a novel pharmacogenetic interaction between WFS1 rs10010131 and urea regulation under SGLT2i therapy. The observed effect could likely reflect a genotype-related renal adaptive response rather than dysfunction, emphasizing the potential of pharmacogenomic profiling to enhance precision treatment for T2D.

## Introduction

Type 2 diabetes (T2D) is a heterogeneous metabolic disorder with marked variation in clinical presentation, disease progression, and treatment response.^1^ While metformin remains the first-line therapy, newer agents such as sodium–glucose cotransporter 2 inhibitors (SGLT2i) have been increasingly adopted due to their cardiovascular and renal benefits.^2^ SGLT2i lower blood glucose by selectively blocking the sodium-glucose cotransporter 2 in the proximal renal tubules, which reduces renal glucose reabsorption and promotes urinary glucose excretion. This insulin-independent mechanism decreases blood sugar levels and also induces sodium excretion, leading to reduced blood pressure and intraglomerular pressure.^3^ These changes contribute to cardiovascular and renal protection.^4^ Additionally, SGLT2i promote weight loss, improve insulin sensitivity, shift metabolism toward ketogenesis, and reduces inflammation and oxidative stress, providing multifaceted benefits beyond glycemic control.^5^,^6^ SGLT2 inhibitors are generally considered to have a satisfactory safety profile,^7^ though they commonly cause increased urination, dehydration, and genital fungal infections, and may also lead to low blood pressure, dizziness, and, in rare cases, serious conditions such as diabetic ketoacidosis and Fournier’s gangrene.^8^

Interindividual differences in treatment outcomes are influenced not only by disease heterogeneity but also by genetic background.^9^,^10^ Advances in pharmacogenomics have highlighted that many common genetic variants can shape drug efficacy and safety, yet the contribution of such variants to intermediate clinical phenotypes in T2D remains underexplored. Genome-wide association studies have identified multiple loci associated with T2D susceptibility and metabolic regulation, but their potential interaction with pharmacological therapies, including SGLT2i, has received limited attention.^11^,^12^ Understanding these treatment–genotype–phenotype relationships could provide important insights into the biology of T2D and support the development of precision medicine strategies.

In this study, we focused on individuals with T2D treated with SGLT2i and employed two comparator groups: patients treated with metformin monotherapy and drug-naïve individuals with T2D, to provide internal validation of observed differences. We systematically evaluated a wide panel of clinical and biochemical traits alongside genetic variants previously implicated in diabetes risk and metabolic pathways. By integrating pharmacological and genetic dimensions, our approach was designed to uncover novel treatment–genotype–phenotype interactions. We hypothesized that pharmacological treatment, particularly SGLT2i use, and common genetic variation jointly contribute to interindividual variability in clinical and biochemical traits among patients with T2D, thereby providing insights into the pharmacogenomic basis of treatment response and metabolic regulation.

## Materials and Methods

### Data Source and Study Participants

**Figure 1:**
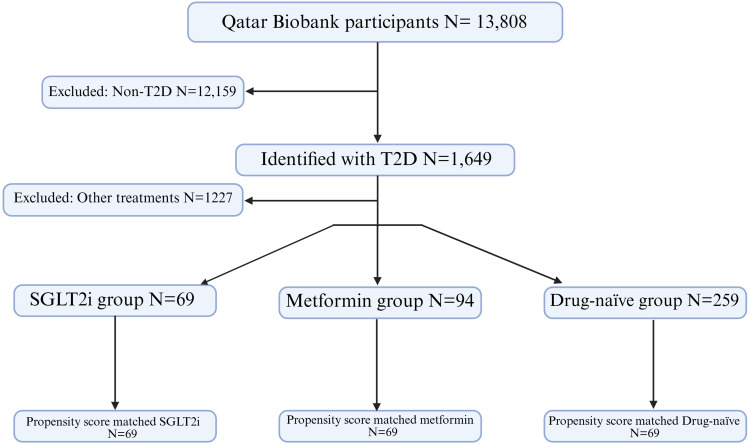
CONSORT flow diagram showing the study design and participant selection.

This study utilized data from 13,808 Qatari nationals and long-term residents recruited by Qatar Biobank (QBB), a population-based prospective initiative by Qatar Foundation, under Qatar Precision Health Institute (QPHI), to promote biomedical research in Qatar and globally.^13^ Data collection included a comprehensive socio-demographic questionnaire along with clinical parameters such as body mass index (BMI), blood pressure, glycated hemoglobin (HbA1c), fasting glucose, insulin levels, lipid profiles, and liver and kidney function markers.^14^ Additional information on medication uses and past medical history, including diabetes, was also recorded.^15^ Among the participants, 1,649 individuals were classified as having type 2 diabetes (T2D) if they met at least one of the following criteria: (i) HbA1c ≥ 6.5%, (ii) fasting glucose ≥ 7 mmol/L, (iii) physician-confirmed diagnosis of diabetes, or (iv) current use of antidiabetic medications. The T2D group was further stratified into three subgroups: The first group (n=69) comprised patients receiving SGLT2i (empagliflozin or dapagliflozin 10 mg), either as monotherapy (10%) or in combination with other anti-diabetics (90%). The second group (n= 94) consisted of patients treated with metformin monotherapy. The third group (n=259) included drug-naïve patients who had not received any pharmacological therapy; these individuals were either newly diagnosed with T2D or were managing their condition exclusively through lifestyle modifications. Additionally, propensity score matching was conducted between the SGLT2i-treated patients and each of the two comparison groups using nearest-neighbor matching with a caliper of 0.2. The matching covariates were age, sex, and BMI. This procedure yielded 69 participants per group, achieving well-balanced characteristics. Participant selection is summarized in the CONSORT flow diagram (Figure 1).

### Genotyping

Whole-genome sequencing of QBB participants was provided by the Qatar Genome project (QGP). Quality control measurements were previously described.^14^ In brief, genomic DNA was extracted using the Qiagen MIDI kit (Qiagen), following the manufacturer’s protocol from participants’ peripheral blood. DNA integrity was measured and quantified using aliper Labchip GXII (Perkin Elmer) Genomic DNA assay and Quant-iT dsDNA Assay (Invitrogen, USA), respectively. Following this, whole-genome libraries were prepared by the Illumina TruSeq DNA Nano kit (Illumina). Genomic libraries were sequenced using HiSeq X Ten (Illumina) with a minimum average coverage of 30X at the Sidra Clinical Genomics Laboratory Sequencing Facility (Sidra Medicine, Qatar). Post-sequencing, quality control was performed using FastQC (v0.11.2) and reads were aligned to the GRCh38 reference genome. Genetic variants associated with variability of SGLT2i effects were identified based on previously published literature.^16–19^ A comprehensive panel of SNPs was selected for analysis (Supplementary Table S1). Genotype counts for each variant across study groups were extracted from WGS data using BCFtools.^20^

### Statistical Analysis

Participants were categorized into genotype groups for each SNP. Data was analyzed using IBM SPSS Statistics for Windows, Version 28.0 (IBM Corp., Armonk, N.Y., USA). GraphPad Prism (GraphPad Software Inc., version 10.1.0) used for visualization. Prior to analysis, all SNPs were tested for deviation from Hardy-Weinberg equilibrium using chi-square tests. Continuous variables are presented as mean ± standard deviation, and categorical variables as frequencies (percentages). Normality of continuous variables was assessed using the Shapiro–Wilk test.

The analysis employed a two-stage approach to identify genotype-phenotype associations specific to SGLT2i treated patients. First, we identified clinical traits that exhibited a significant differential effect across treatment groups (SGLT2i treated patients vs. metformin treated patients and drug-naïve individuals). Only these traits were advanced to the genetic association analysis.

In the second stage, for each of these selected traits, we tested for associations with genotype within the SGLT2i user group. This was performed using one-way ANOVA for each SNP, with genotype as the independent variable and the clinical trait as the dependent variable. Assumptions of homogeneity of variance were verified using Levene’s test. For SNPs yielding a significant result, post-hoc pairwise comparisons were conducted with Bonferroni correction.

SNP–trait pairs that showed a significant association in the ANOVA were subsequently analyzed using multiple linear regression to adjust for potential confounders. For these analyses, we specified an additive genetic model for each SNP, coding genotypes by the number of effect alleles (0, 1, or 2), which is standard for common variants and consistent with the graded genotype–trait patterns observed. Regression assumptions, including linearity, independence of errors, homoscedasticity, and normality of residuals, were verified. Results are reported as beta coefficients with 95% confidence intervals (CIs). A significance threshold of p < 0.05 was applied, with adjustment for multiple testing across SNPs for a given trait. To determine whether the identified genetic associations were specific to the SGLT2i treatment context, we tested for genotype-by-treatment interaction. Planned contrasts were embedded within the regression framework. These contrasts directly compared the magnitude of the genotype effect in the SGLT2i user group against the combined average effect in the metformin and drug-naïve groups. We also calculated the linkage disequilibrium (LD) between variants using QGP genotype data in PLINK. LD metric including r2 and D’ were estimated to assess the genetic linkage relationship between these loci. Expression quantitative trait locus (eQTL) analysis was performed using GTEx v8 single-tissue gene expression data portal. To examine tissue-specific expression levels, we obtained associations for the SNPs of interest, focusing primarily on tissues relevant to diabetes pathogenesis. Post hoc power calculations were performed for the primary pharmacogenetic associations. As a sensitivity analysis, we additionally fitted multivariable models for serum urea including treatment group and further adjustment for key metabolic and renal covariates within the propensity score-matched cohort, to evaluate the robustness of the SGLT2i-urea association to residual confounding.

## Results

### General Characteristics of Participants

We employed propensity score matching techniques to balance the demographic characteristics of age, BMI, and sex between groups. As a result of this procedure, the final sample consisted of 207 participants, evenly distributed with 69 individuals in each group.

The comparative analysis (Table 1) identified five clinical parameters that were significantly different in the SGLT2i user group compared to both the metformin and drug-naïve groups (FDR < 0.05 for both comparisons). The results include expected changes in lipid profiles (total and LDL-cholesterol), uric acid, and hematocrit, which are consistent with the known pleiotropic effects of this drug class.^21^,^22^ However, the high and significant serum urea levels in the SGLT2i-treated patients compared to both control groups represents a novel and less-explored finding. This observation prompted a focused investigation to determine if genetic variation influences urea levels specifically within the context of SGLT2i treatment. Moreover, in the sensitivity analysis adjusting for additional metabolic and renal covariates, the higher urea levels in SGLT2i-treated patients versus metformin and drug-naive groups remained directionally consistent and of similar magnitude, supporting the robustness of this association to measured metabolic differences. Consequently, urea was selected for in-depth genetic association analysis.

**Table 1:** Demographic Characteristics of Participants

Variable	G1 SGLT2i Treated Patients N=69	G2 Metformin Treated Patients N=69	G3 Drug-Naïve N=69	G1 vs G2 FDR	G1 vs G3 FDR
Sex (Male/Female)	26/43	24/45	25/44	0.9	0.9
Age	54 (48–60)	53 (48–57)	54 (47–61)	0.8	0.9
BMI (kg/m^2^)	31.4 (26.8–34.4)	31.6 (27.4–36.0)	30.7 (28.2–34.9)	0.28	0.23
Fasting blood glucose (mmol/L)	7.6 (6.2–9.3)	7.1 (5.8–9.7)	6.6 (6–8.7)	0.72	0.56
HbA_1_C (%)	7.8 (7–8.7)	6.8 (6.2–8.4)	7 (6.6–7.4)	0.04	0.11
Insulin (uU/mL)	12.4 (7.3–22.2)	10.4 (7.22–17.03)	14.5 (8.75–21.25)	0.19	0.747
C-Peptide	2.29 (1.66–3.01)	2.34 (1.81–3.05)	3 (2.11–4.06)	0.49	0.014
Total cholesterol (mmol/L)	4.59 (0.94)	5.15 (0.93)	5.59 (1.06)	0.013	p<0.001
HDL-cholesterol (mmol/L)	1.3 (1.04–1.6)	1.25 (1–1.5)	1.3 (1.1–1.6)	0.56	0.899
LDL-cholesterol (mmol/L)	2.51 (0.87)	3.12 (0.87)	3.41 (0.99)	0.004	p<0.001
Triglyceride (mmol/L)	1.4 (1.1–1.8)	1.6 (1.2–1.8)	1.63 (1.2–2.2)	0.62	0.33
Creatinine (µmol/L)	66 (54–75)	60 (52–70)	62 (54–75)	0.64	0.93
Urea (mmol/L)	5.1 (4.4–6)	4.2 (3.4–4.8)	4.5 (3.5–5)	0.002	0.01
Uric acid	253 (201–286)	279 (242.75–332)	308 (267–359)	0.047	p<0.001
Albumin	41.19 (3.75)	42.78 (3.99)	41.47 (4.19)	0.081	0.81
Total protein	74 (72–77)	73 (71–76)	75 (72–78)	0.32	0.52
Sodium	140.12 (2.29)	139.77 (2.17)	140.07 (2.68)	0.71	0.95
Potassium	4.52 (0.42)	4.38 (0.31)	4.45 (0.37)	0.18	0.51
Chloride	101 (99–103)	100 (99–102)	100 (99–102)	0.64	0.4
Calcium	2.41 (0.09)	2.39 (0.1)	2.37 (0.09)	0.51	0.11
ALT	20 (14–28)	20 (14–24)	22 (17–32)	0.78	0.22
AST	18 (14–23)	16 (14–22)	19 (16–24)	0.85	0.11
Hematocrit	42.23 (4.55)	40.03 (4.16)	40.18 (4.75)	0.032	0.032

**Notes**: Data are expressed as mean (standard deviation) for variables with normal distribution and as median (interquartile range) for non-normally distributed variables, as determined by the Shapiro–Wilk test. Comparisons between SGLT2i-treated patients and each of the other 2 groups were conducted using either Student’s *t*-test or the Mann–Whitney *U*-test, as appropriate. A p-value < 0.05 was considered statistically significant.

### Genetic Association with Serum Urea Levels

**Figure 2:**
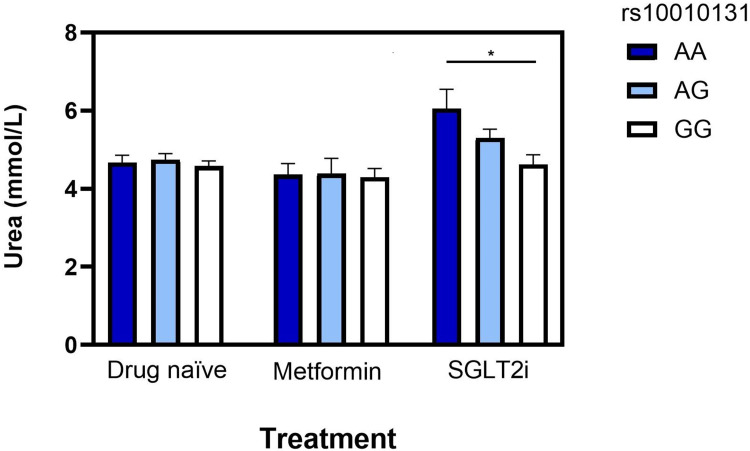
Serum urea levels (mmol/L) are shown across the three treatment groups: drug-naïve, metformin-treated, and SGLT2i-treated. Within each group, data are further stratified by the rs10010131 genotype (AA, AG, GG). Data are presented as mean ± SEM. *p < 0.01 for post-hoc comparisons between genotype groups within the SGLT2i cohort after one-way ANOVA.

Preliminary ANOVA with Bonferroni post-hoc testing indicated a significant difference in mean urea levels across the ss10010131 genotype groups (Figure 2). Interestingly, we observed that rs10010131 genotype stratified urea levels in SGLT2i treated patients, with AA carriers having the highest levels, GG the lowest, and AG showing intermediate values. Strikingly, this genotype-dependent trend was not observed in metformin or drug-naïve groups (Figure 2), pointing toward a specific gene–drug interaction. The linear regression model incorporating the planned contrast, adjusted for age, sex, and BMI corroborated our results. The association between the rs10010131 A allele and increased urea was significantly stronger in the SGLT2i-treated patients compared to the average effect in the metformin and drug-naive control groups (p <0.001).

To adjust for potential confounding variables, the association was further analyzed using a multiple linear regression model with an additive genetic term while adjusting for age, sex and BMI as covariates. After adjusting for age, sex, and BMI, each additional A allele was associated with a significant increase of 0.63 mmol/L (95% CI [0.14,1.11]; p= 0.012) in serum urea levels (Table 2). This suggests that each “A” allele contributes to a dose-dependent increase in urea levels in the context of SGLT2i use. Given the modest sample size of the pharmacogenetic analysis, we conducted a post hoc power analysis for the rs10010131–urea association within SGLT2i treated patients (n 69). Based on the observed per-allele effect, the achieved power to detect an effect of this magnitude at a two-sided α of 0.05 was approximately 75%, indicating moderate power for the detected association but limited sensitivity for smaller genetic effects.

**Table 2:** Multiple Linear Regression Analysis of Genetic and Clinical Factors Associated with Serum Urea Levels in SGLT2i-Treated Patients

Predictor	Beta	95% CI	P
A allele of rs10010131	+0.63	[0.14, 1.11]	0.012
Age	+0.02	[−0.02, 0.06]	0.24
Sex	−0.72	[−1.55, 0.76]	0.08
BMI	+0.032	[−0.33, 0.09]	0.33

## Discussion

In this study, we compared the clinical characteristics of T2D patients treated with SGLT2i, metformin, or no medication. The unanticipated high levels of urea in the SGLT2i-treated patients prompted a genetic investigation to determine if common variants associated with drug response could explain this variability, thereby translating a population-level observation into a potential insight for personalized medicine. Our study identifies a novel and significant pharmacogenetic interaction where the A allele of the rs1001013 in the *WFS1* gene is associated with elevated serum urea levels specifically in T2D patients treated with SGLT2i. This finding was not observed in metformin-treated or drug-naïve individuals, suggesting a unique gene-drug interaction rather than a direct effect of the genotype on urea metabolism.

*WFS1* encodes wolframin, a glycoprotein primarily localized in the endoplasmic reticulum (ER) membrane. Wolframin is essential for proper ER function and cell survival in tissues critical for glucose regulation and neuroendocrine health, as it regulates ER calcium homeostasis and ER stress response. It is ubiquitously expressed but particularly abundant in pancreatic beta cells and brain. *WFS1* variants cause elevated ER stress, activation of the unfolded protein response, impaired insulin processing and secretion, and apoptosis in neurons and beta cells. These changes underlie what is called Wolfram syndrome and related diabetes phenotypes.^23^

The rs10010131 variant has been previously implicated in renal and metabolic traits, though in different contexts. For instance, Franceschini et al^24^ identified in their meta-analysis that rs10010131G was linked to higher estimated glomerular filtration rate (eGFR) in younger individuals (under 45 years) and increased albuminuria, which are early markers of diabetic nephropathy. This SNP’s effect on eGFR was age-dependent, showing a much stronger association in younger participants. Interestingly, the A allele of rs10010131 is shown to be strongly associated with a reduced risk of T2D, consistently showing a protective effect across multiple large studies, highlighting its role in pancreatic beta-cell function and endoplasmic reticulum stress response.^25^

The relation of rs10010131 in the context of SGLT2i treatment was investigated by Pereira et al^26^ They identified a significant association between the rs10010131A and bodyweight loss among participants treated with a combination of dapagliflozin and once weekly exenatide. They reported that each additional copy of the A allele corresponded to an approximate 2.4 kg greater reduction in bodyweight. Their results suggest that rs10010131 may serve as a valuable genetic marker for predicting enhanced weight loss response to dapagliflozin and exenatide therapy in obese individuals without diabetes. However, the absence of monotherapy arms in the study limits the ability to draw definitive conclusions regarding the association between the tested variant and SGLT2i treatment response.

SGLT2i is not known to affect urea levels. Scholtes et al^27^ demonstrated that during two weeks of dapagliflozin treatment in people with type 2 diabetes, mean serum urea levels remained stable over time. The association between the rs10010131 A allele and elevated urea levels in SGLT2i-treated patients in our study invites a cautious interpretation. In the negative frame, the *WFS1* variant may confer a latent vulnerability in renal tubular cells, likely by impairing ER stress responses, which is unmasked by the solute diuresis of SGLT2 inhibition. However, the fact that A allele carriers maintained comparable glycemic control and creatinine levels (Supplementary Table S2) powerfully counters the notion of a general adverse effect, as it dissociates the urea finding from both deteriorating metabolic function and a decline in glomerular filtration rate. This specific biochemical profile gives greater weight to a positively framed hypothesis that the urea elevation is likely a biomarker of a more robust and beneficial adaptive response. The A allele, already linked to a reduced risk of diabetes,^25^ might facilitate a superior hemodynamic adaptation to SGLT2i. The higher urea could thus indicate a more effective activation of tubuloglomerular feedback and a stronger restoration of the medullary osmotic gradient for water conservation which are the central mechanisms behind the drugs’ proven cardiorenal benefits.^28^,^29^ In this scenario, the genotype identifies a superior responder profile, where the physiological changes that underpin long-term organ protection are simply more pronounced. The critical task for future research is to resolve this ambiguity by determining whether this genetic interaction ultimately predicts an adverse renal trajectory or, more optimistically, a uniquely favorable long-term outcome.

**Figure 3:**
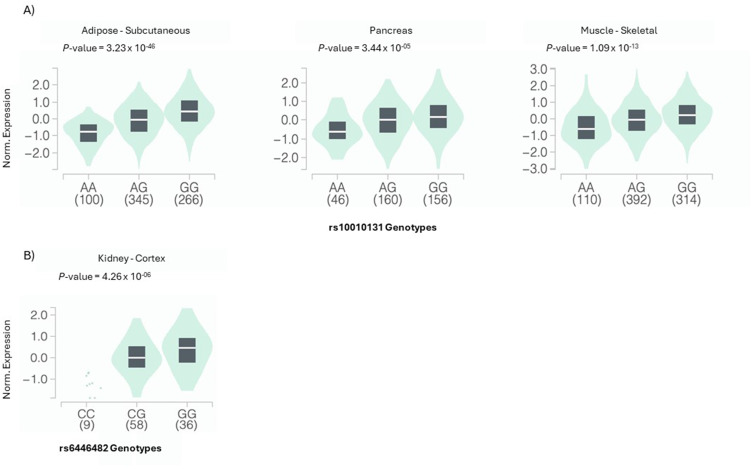
Box-and-whisker plots show WFS1 expression levels stratified by rs10010131 and rs6446482 genotype. The SGLT2i-associated SNP rs10010131 is a significant eQTL for WFS1 in (**A**) subcutaneous adipose tissue (P = 3.23 × 10^−46^), pancreas (P = 3.44 × 10^−5^), and skeletal muscle (P = 1.09 × 10^−13^). (**B**) rs6446482 is a significant eQTL for WFS1 expression in kidney cortex (P = 4.26 × 10^−6^).

Moreover, our results demonstrate an exceptionally strong linkage disequilibrium (LD) between rs10010131 and rs6446482, a second variant located within the same *WFS1* gene but not directly examined in this study, for which potential LD was assessed. The high LD metrics (r^2^ = 0.95, D′ = 0.99) are consistent with previous reports,^30^,^31^ indicating that genotype classifications for rs6446482 can serve as reliable proxies for rs10010131 genotypes in genetic analyses and interpretation. Importantly, the clear, dose-dependent increase in urea levels across the rs6446482 genotype groups (CC to GG) specifically within the SGLT2i-treated cohort (Supplementary Figure S1) robustly reflects the effect of the rs10010131 A allele-associated haplotype. Interestingly, functional studies showed that the kidney-specific eQTL for rs6446482 (Figure 3) has a very reduced expression of *WFS1* in the CC genotype. This could be explained by the fact that the haplotype carrying the A and C alleles at rs10010131 rs6446482 respectively appears to downregulate the expression of the *WFS1* gene in the renal cortex. This establishes a compelling genotype-to-phenotype pathway, wherein the genetic variant associated with high urea is a proxy for lower renal wolframin levels. We therefore posit that a pre-existing state of reduced wolframin in the renal tubules, a key regulator of ER stress, creates a unique cellular environment that, under SGLT2i challenge, leads to the pronounced, treatment-specific elevation in urea observed in our data. Importantly, this mechanistic interpretation is inferred from prior functional studies of WFS1 and SGLT2i physiology rather than directly demonstrated in our cohort, and should therefore be regarded as a hypothesis-generating framework rather than a proven causal pathway.

In interpreting our findings, several limitations should be acknowledged. First, the study is cross-sectional and observational, with urea levels and treatment status captured at a single time point. As a result, temporal changes in urea before and after initiation of SGLT2i therapy were not assessed, and we cannot determine whether the observed elevation in urea reflects a treatment-induced change, pre-existing differences in patients selected for SGLT2i, or unmeasured confounding. Consequently, neither the association between SGLT2i use and higher urea levels nor the interaction between rs10010131 and SGLT2i exposure can be interpreted as causal, and our results should be viewed as hypothesis-generating rather than proof of a causal drug–gene effect. Future studies with prospective, longitudinal designs, including pre- and post-treatment sampling and explicit genotype–treatment stratification, will be required to establish whether rs10010131 truly modifies urea trajectories or renal outcomes under SGLT2i therapy.

Second, the statistical power for genetic association analyses is limited. Although the propensity-matched design yielded a well-balanced SGLT2i-treated group, this sample size is modest for pharmacogenomic investigation, particularly when broken down by genotype strata. A post hoc power calculation based on the observed per-allele effect of rs10010131 on urea in SGLT2i treated patients indicated only moderate power to detect an effect of this magnitude at a two-sided α of 0.05, and substantially lower power for smaller genetic effects. Further research is warranted replicate this pharmacogenetic signal in larger, independent and ethnically diverse cohorts.

## Conclusion

In conclusion, our study identifies a novel pharmacogenetic interaction where the rs10010131 A allele in the *WFS1* gene is associated with elevated serum urea levels specifically in T2D patients treated with SGLT2i. This finding highlights the importance of genetic background in modulating specific metabolic responses to anti-diabetic drugs. It suggests that a patient’s genotype could influence their laboratory profile during treatment, which may have implications for monitoring and personalized management. Future prospective studies are warranted to elucidate the underlying biological mechanism and to explore the potential clinical relevance of this drug-gene interaction on long-term renal outcomes.

## Data Availability

The datasets used and/or analyzed during the current study are available from the corresponding author on reasonable request.

## References

[1] Suzuki K, Hatzikotoulas K, Southam L, et al. Genetic drivers of heterogeneity in type 2 diabetes pathophysiology. *Nature*. 2024;627(8003):347–9. doi:10.1038/s41586-024-07019-6 38374256 PMC10937372

[2] Rabizadeh S, Nakhjavani M, Esteghamati A. Cardiovascular and renal benefits of SGLT2 inhibitors: a narrative review. *Int J Endocrinol Metab*. 2019;17(2):e84353. 31372172 10.5812/ijem.84353 PMC6628616

[3] Fonseca-Correa JI, Correa-Rotter R. Sodium-Glucose cotransporter 2 inhibitors mechanisms of action: a review. *Front Med*. 2021;8:777861. doi:10.3389/fmed.2021.777861 PMC8720766 34988095

[4] Vallon V, Verma S. Effects of SGLT2 inhibitors on kidney and cardiovascular function. *Annu Rev Physiol*. 2021;83(1):503–528. doi:10.1146/annurev-physiol-031620-095920 33197224 PMC8017904

[5] Pereira MJ, Eriksson JW. Emerging role of SGLT-2 inhibitors for the treatment of obesity. *Drugs*. 2019;79(3):219–230. doi:10.1007/s40265-019-1057-0 30701480 PMC6394798

[6] Hasan I, Rashid T, Jaikaransingh V, et al. SGLT2 inhibitors: beyond glycemic control. *J Clin Translat Endocrinol*. 2024;35:100335. doi:10.1016/j.jcte.2024.100335 PMC10957445 38525377

[7] Scheen AJ. An update on the safety of SGLT2 inhibitors. *Expert Opin Drug Saf*. 2019;18(4):295–311. doi:10.1080/14740338.2019.1602116 30933547

[8] McLean P, Bennett J, Trey Woods E, et al. SGLT2 inhibitors across various patient populations in the era of precision medicine: the multidisciplinary team approach. *Npj Metabolic Health Dis*. 2025;3(1):29. doi:10.1038/s44324-025-00068-z

[9] Dhieb D, Mustafa D, Hassiba M, et al. Harnessing pharmacomultiomics for precision medicine in diabetes: a comprehensive review. *Biomedicines*. 2025;13(2):447. doi:10.3390/biomedicines13020447 40002860 PMC11853021

[10] Ordelheide AM, Hrabě de Angelis M, Häring H-U, et al. Pharmacogenetics of oral antidiabetic therapy. *Pharmacogenomics*. 2018;19(6):577–587. doi:10.2217/pgs-2017-0195 29580198

[11] Elashi AA, Toor SM, Umlai U-KI, et al. Genome-wide association study and trans-ethnic meta-analysis identify novel susceptibility loci for type 2 diabetes mellitus. *BMC Med Genomics*. 2024;17(1):115. doi:10.1186/s12920-024-01855-1 38685053 PMC11059680

[12] Xue A, Wu Y, Zhu Z, et al. Genome-wide association analyses identify 143 risk variants and putative regulatory mechanisms for type 2 diabetes. *Nat Commun*. 2018;9(1):2941. doi:10.1038/s41467-018-04951-w 30054458 PMC6063971

[13] Al Thani A, Fthenou E, Paparrodopoulos S, et al. Qatar Biobank Cohort Study: study design and first results. *Am J Epidemiol*. 2019;188(8):1420–1433. doi:10.1093/aje/kwz084 30927351

[14] Thareja G, Al-Sarraj Y, Belkadi A, et al. Whole genome sequencing in the Middle Eastern Qatari population identifies genetic associations with 45 clinically relevant traits. *Nat Commun*. 2021;12(1):1250. doi:10.1038/s41467-021-21381-3 33623009 PMC7902658

[15] Suhre K, Stephan N, Zaghlool S, et al. Matching drug metabolites from non-targeted metabolomics to self-reported medication in the Qatar Biobank Study. *Metabolites*. 2022;12(3):249. doi:10.3390/metabo12030249 35323692 PMC8948833

[16] Saravana Kumar P, Chidambaram Y, Shree Devi G, et al. Genomic insights about the effect of sodium-glucose cotransporter 2 inhibitors: a systematic review. *Front Genetics*. 2025;16. doi:10.3389/fgene.2025.1571032 PMC12162637 40520225

[17] Klen J, Dolžan V. Treatment response to SGLT2 inhibitors: from clinical characteristics to genetic variations. *Int J Mol Sci*. 2021;22(18):9800. doi:10.3390/ijms22189800 34575958 PMC8466905

[18] Imamovic Kadric S, Kulo Cesic A, Dujic T. Pharmacogenetics of new classes of antidiabetic drugs. *Bosn J Basic Med Sci*. 2021;21(6):659–671. doi:10.17305/bjbms.2021.5646 33974529 PMC8554705

[19] Xu B, Li S, Kang B, et al. The current role of sodium-glucose cotransporter 2 inhibitors in type 2 diabetes mellitus management. *Cardiovascular Diabetol*. 2022;21(1):83. doi:10.1186/s12933-022-01512-w PMC9134641 35614469

[20] Danecek P, Bonfield JK, Liddle J, et al. Twelve years of SAMtools and BCFtools. *Gigascience*. 2021;10(2). 10.1093/gigascience/giab008 PMC7931819 33590861

[21] Zhang L, Zhang F, Bai Y, et al. Effects of sodium-glucose cotransporter-2 (SGLT-2) inhibitors on serum uric acid levels in patients with chronic kidney disease: a systematic review and network meta-analysis. *BMJ Open Diabetes Res Care*. 2024;12(1):e003836. 10.1136/bmjdrc-2023-003836 PMC10807021 38238025

[22] Kanbay M, Tapoi L, Ureche C, et al. Effect of sodium-glucose cotransporter 2 inhibitors on hemoglobin and hematocrit levels in type 2 diabetes: a systematic review and meta-analysis. *Int Urol Nephrol*. 2022;54(4):827–841. doi:10.1007/s11255-021-02943-2 34273060

[23] Li Y, Gong S, Li M, et al. The genetic and clinical characteristics of WFS1 related diabetes in Chinese early onset type 2 diabetes. *Sci Rep*. 2023;13(1):9127. doi:10.1038/s41598-023-36334-7 37277527 PMC10241780

[24] Franceschini N, Shara NM, Wang H, et al. The association of genetic variants of type 2 diabetes with kidney function. *Kidney Int*. 2012;82(2):220–225. doi:10.1038/ki.2012.107 22513821 PMC3664521

[25] Cheng S, Wu Y, Wu W, et al. Association of rs734312 and rs10010131 polymorphisms in WFS1 gene with type 2 diabetes mellitus: a meta-analysis. *Endocr J*. 2013;60(4):441–447. doi:10.1507/endocrj.EJ12-0325 23257691

[26] Pereira MJ, Lundkvist P, Kamble PG, et al. A randomized controlled trial of dapagliflozin plus once-weekly exenatide versus placebo in individuals with obesity and without diabetes: metabolic effects and markers associated with bodyweight loss. *Diabetes Ther*. 2018;9(4):1511–1532. doi:10.1007/s13300-018-0449-6 29949016 PMC6064580

[27] Scholtes RA, Muskiet MHA, van Baar MJB, et al. The adaptive renal response for volume homeostasis during 2 weeks of dapagliflozin treatment in people with type 2 diabetes and preserved renal function on a sodium-controlled diet. *Kidney Int Rep*. 2022;7(5):1084–1092. doi:10.1016/j.ekir.2022.02.023 35570989 PMC9091605

[28] Salvatore T, Galiero R, Caturano A, et al. An Overview of the Cardiorenal Protective Mechanisms of SGLT2 Inhibitors. *Int J Mol Sci*. 2022;23(7). 10.3390/ijms23073651 PMC8998569 35409011

[29] Dai Z-C, Chen J-X, Zou R, et al. Role and mechanisms of SGLT-2 inhibitors in the treatment of diabetic kidney disease. *Front Immunol*. 2023;14. doi:10.3389/fimmu.2023.1213473 PMC10552262 37809091

[30] Sandhu MS, Weedon MN, Fawcett KA, et al. Common variants in WFS1 confer risk of type 2 diabetes. *Nat Genet*. 2007;39(8):951–953. doi:10.1038/ng2067 17603484 PMC2672152

[31] Florez JC, Jablonski KA, McAteer J, et al. Testing of diabetes-associated WFS1 polymorphisms in the diabetes prevention program. *Diabetologia*. 2008;51(3):451–457. doi:10.1007/s00125-007-0891-x 18060660 PMC2483955

